# Cure of Meniere’s Disease

**Published:** 1880-04-20

**Authors:** J. T. Jenkins

**Affiliations:** Kentucky


### *CURE OF MENIERE'S DISEASE.*

BY J. T. JENKINS, M.D., OF KENTUCKY.

*Editor of Record.*—DEAR SIR: The following case may be of interest to the profession.

Mr. J. D., aged 40; large, stout, muscular; former general health good; takes a drinking spree occasionally; has been having what he and his family call fits. Falls suddenly without warning; does not lose consciousness; feels a little giddy for a few minutes after the attacks.

After a careful examination of him I was unable to find any cause for his trouble until my attention was called to the condition of his left ear, in which he complained of a slight buzzing noise with some tenderness below and back of ear. On making specular examination I found the cavity nearly filled with hardened secretions of wax. He dates the vertigo from the treatment he received from a physician who insufflated the tympanic cavity.

After a thorough cleaning of ear with warm water and soap, with blisters behind ears, the spells have not returned, now over three months.

My object in reporting the above case is that I suppose it was a case of Meniere's disease; a report of the conclusions of Dr. Guge's paper in *Southern Medical Record* for January 1880, on that disease, explains what would have been very obscure to me without the information thus gained.

